# When a Drain is the Culprit: An Unexpected Case of Small Bowel Obstruction with Biliary Peritonitis

**DOI:** 10.7759/cureus.4964

**Published:** 2019-06-21

**Authors:** Maher Al Khaldi, Félix Thibeault, Richard Létourneau

**Affiliations:** 1 General Surgery, Centre Hospitalier de l'Université de Montréal (CHUM), Montreal, CAN; 2 General Surgery, Centre Hospitalier de l’Université de Montréal (CHUM), Montreal, CAN; 3 Pancreatic and Hepatobiliary Surgery, Centre Hospitalier de l’Université de Montréal (CHUM), Montreal, CAN

**Keywords:** drains, bowel obstruction, cholangiocarcinoma, biliary peritonitis

## Abstract

Although postoperative abdominal drains are useful in therapeutic settings, their prophylactic role is debatable. We herein describe the case of a 30-year-old male who underwent bile duct resection with hepaticojejunostomy for cholangiocarcinoma. On postoperative day four, the patient developed biliary peritonitis. Explorative laparotomy revealed an obstruction of the afferent limb caused by an intestinal loop around a Jackson-Pratt (JP) drain. Removal of the drain resolved the obstruction which led to a significant improvement of the patient’s clinical state. To the best of our knowledge, this is the second report of a bowel obstruction from a surgical drain. When placing abdominal drains, surgeons must take into consideration their indication as well as possible related complications, including intestinal obstruction.

## Introduction

Prophylactic intra-abdominal drain placement following surgery aims at draining intraperitoneal collections and reducing the risk of subsequent infection [1]. However, the value of prophylactic drains has been debated for the past decades [2]. Growing evidence is showing no added benefit to prophylactic abdominal drainage, but drain placement may also increase patient morbidity [3]. Several complications include infection, bleeding, and tissue erosion. The following report describes a case of intestinal obstruction caused by a Jackson-Pratt (JP) drain, a very uncommon complication that is unknown to many practitioners. 

## Case presentation

A 30-year-old male with no significant past medical or surgical history presented with cholestatic jaundice. An abdominal ultrasonography (USG) revealed an 11-mm obstructive lesion in the common bile duct with intrahepatic duct dilatation. An attempt at stent placement to relieve jaundice failed during endoscopic retrograde cholangiopancreatography (ERCP). A magnetic resonance cholangiopancreatography (MRCP) subsequently showed no gallstones but revealed a 7.0 mm segmental stenosis of the common hepatic duct with intrahepatic bile duct dilatation. The lesion was further characterized with an endoscopic ultrasonography (EUS), revealing a 19.0 mm lesion at the common hepatic duct, suspicious for a cholangiocarcinoma (Klatskin tumor). Thoracic, abdominal, and pelvic scans revealed no evidence of metastatic disease and no vascular involvement. The tumour was therefore deemed resectable. 

**Figure 1:**
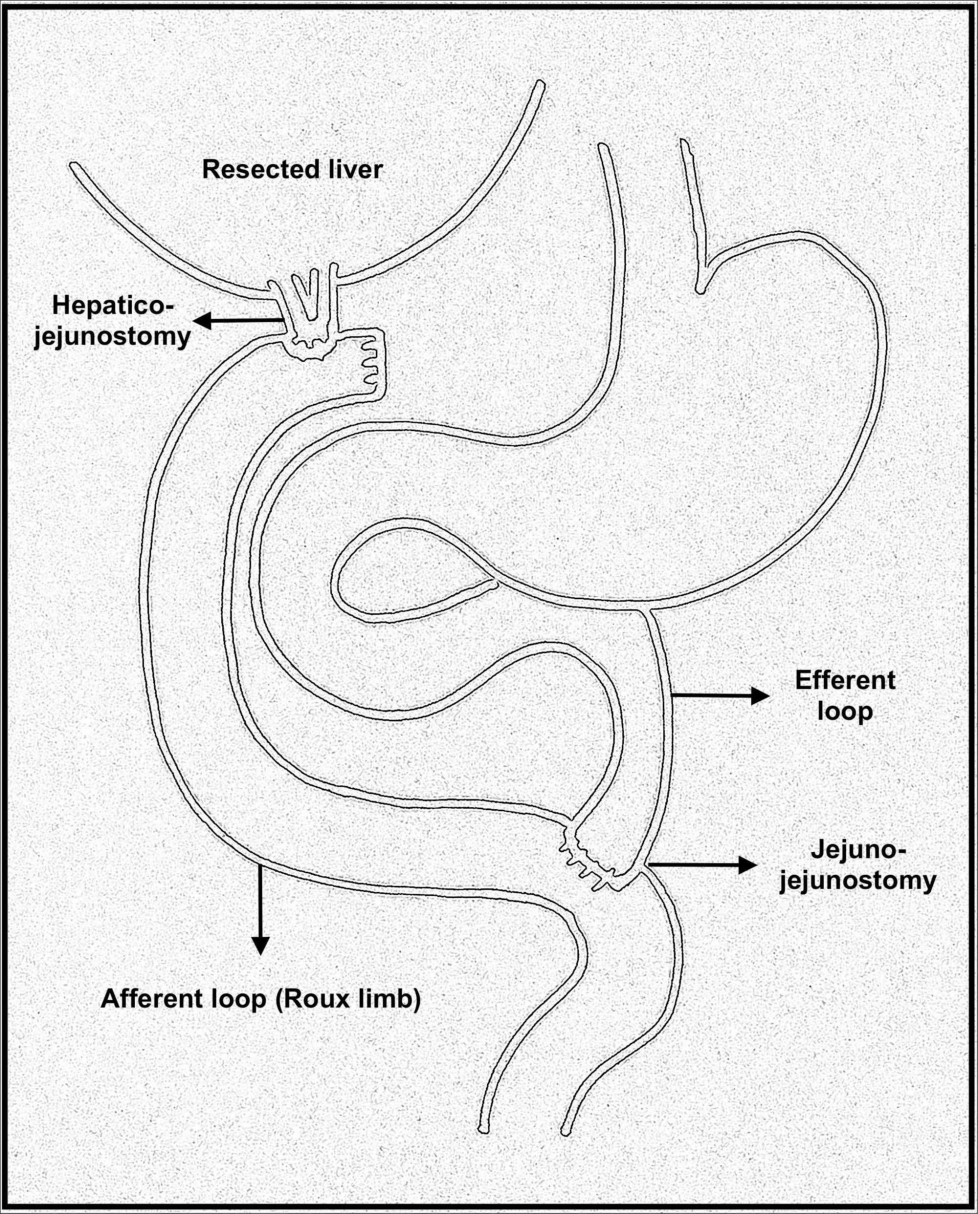
A sketch depicting a Roux-en-Y hepatico-jejunostomy.

The patient underwent an extended right hepatectomy, cholecystectomy, extrahepatic duct resection, and lymphadenectomy, followed by a Roux-en-Y hepaticojejunostomy (Figure 1).

Two prophylactic JP drains were placed at the right upper quadrant around the anastomoses to monitor for postoperative anastomotic leaks. The procedure was successfully performed and well tolerated by the patient without any immediate post-operative complications.

The pathology report later confirmed a perihilar extrahepatic bile duct adenocarcinoma. No evidence of lymph node involvement or metastatic disease was found.

On postoperative day four, the patient became febrile and complained of abdominal pain and vomiting. Physical examination revealed diffuse rebound tenderness was present, with maximal tenderness at the upper right quadrant. Bile was noted in both JP drains. 

Elevated cholestatic markers were noted on laboratory results. An urgent abdominal CT scan revealed a severe dilation of the proximal jejunum (up to 5.2 cm) with a possible transition point at the level of the proximal jejunum. A hypodense zone suggesting a perianastomotic collection of 5.0 cm x 3.0 cm was noted. The radiological images suggested an anastomotic leak secondary to the obstruction of the afferent intestinal loop, making biliary peritonitis the most likely diagnosis. 

**Figure 2:**
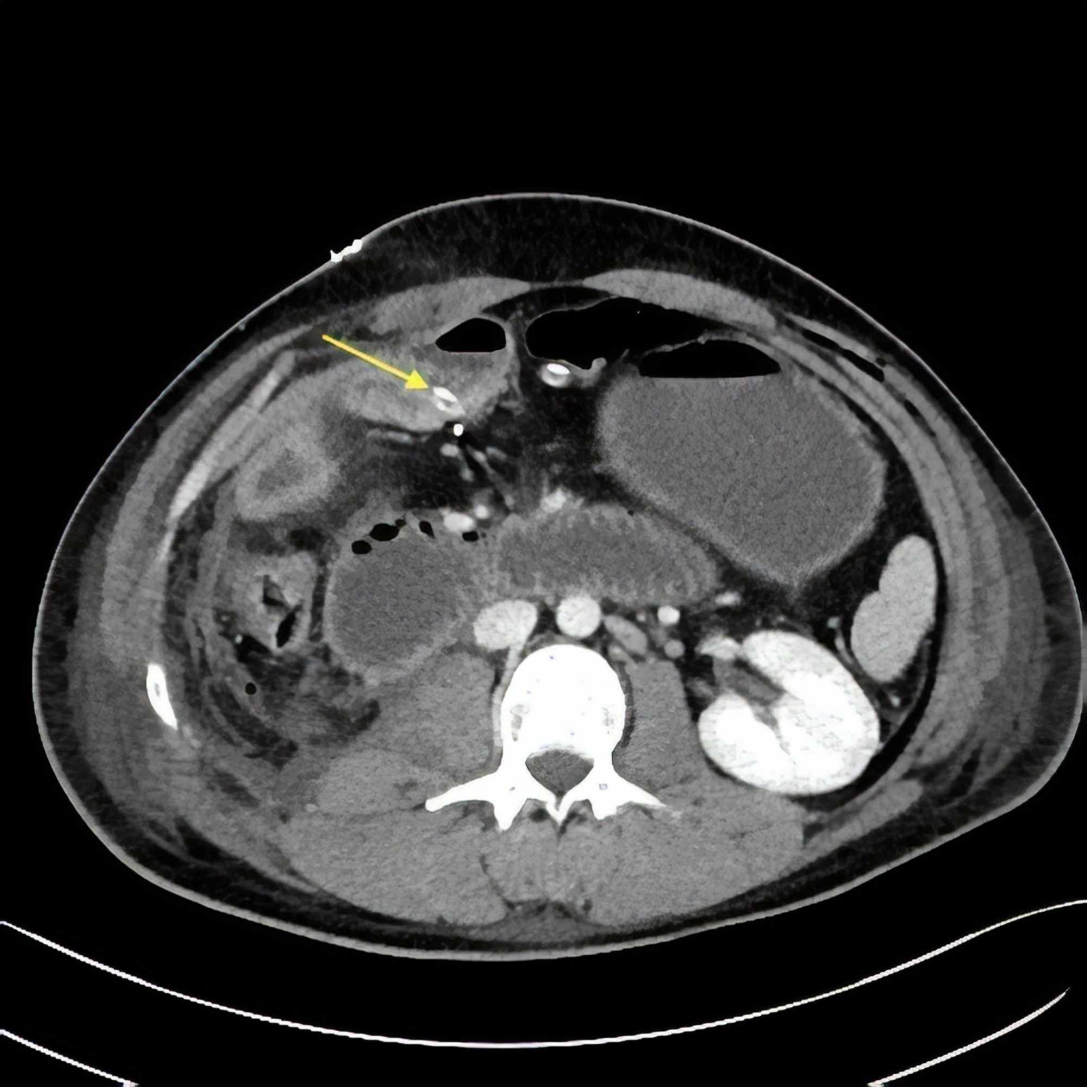
Axial view of the CT scan depicting the transition point of the obstruction close to the right abdominal Jackson-Pratt (JP) (arrow).

**Figure 3:**
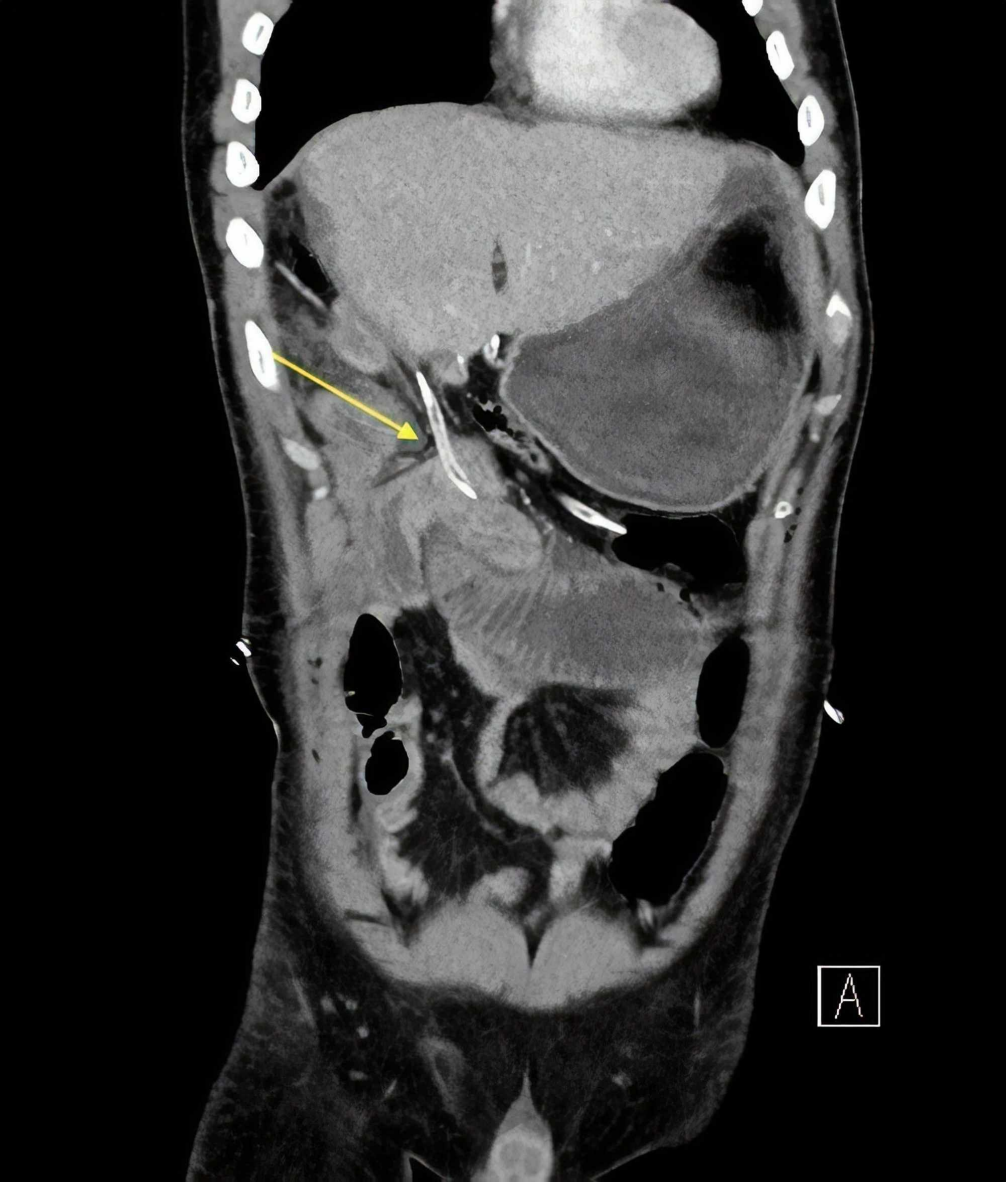
Coronal view of the CT scan showing the trajectory of the culprit drain. The stomach is distended as a result of the obstruction.

**Figure 4:**
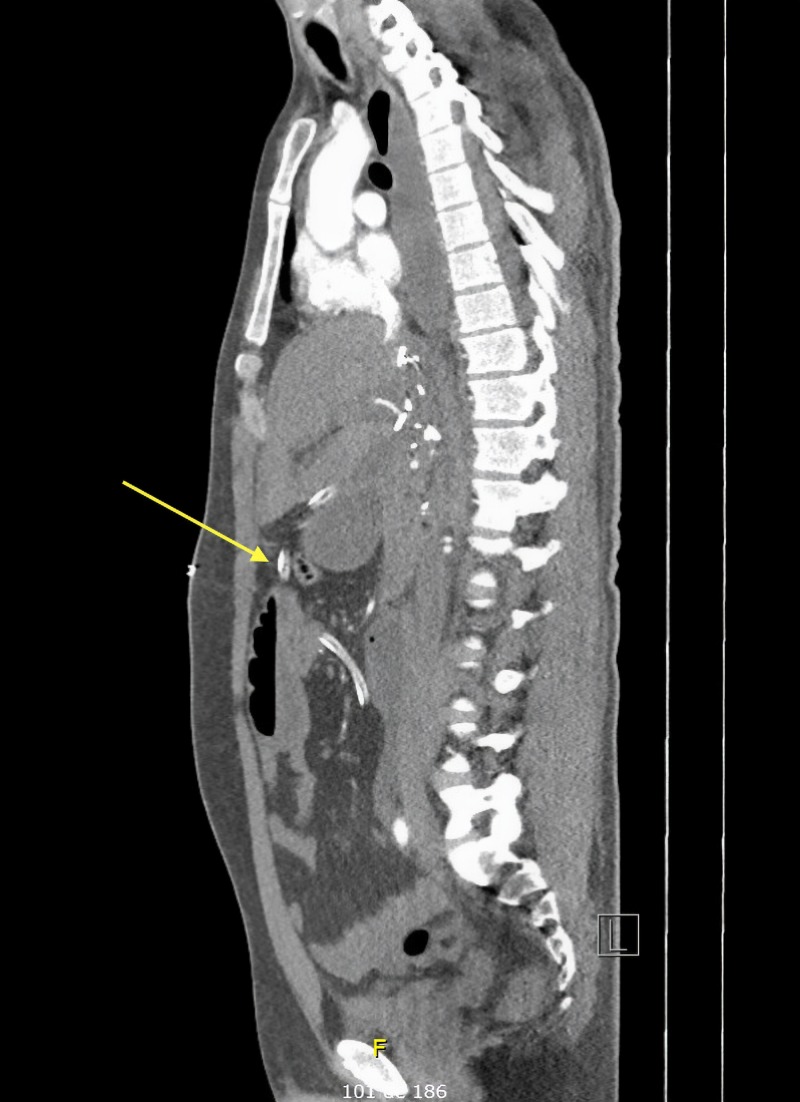
Sagittal view of the CT scan showing, once again, the drain’s course throughout the abdomen.

The patient underwent urgent exploratory laparotomy. A significant intestinal distention was noted upon opening of the abdomen. Surprisingly, the proximal jejunum was looped around one of the JP drains close to the hepaticojejunostomy (Figures 2-4). 

Removal of the drain led to the spontaneous resolution of the obstruction and resulted in normal drainage of the intestinal content into the distal intestine. 

The patient was hospitalized for several weeks due to multiple complications, including gastroparesis and transient hepatic insufficiency. After significant clinical improvement, the patient was discharged from the hospital with a closely scheduled follow-up appointment.

## Discussion

The use of surgical drains has been practiced for many years and was even described in the era of Hippocrates for drainage of empyema and ascites [4]. In the 19th century, the German surgeon Theodore Billroth supported prophylactic drainage after gastrointestinal surgery, claiming it led to better clinical outcome [5]. 

Prophylactic drains address intraperitoneal collections such as bile, blood, ascites and intestinal or pancreatic secretions. The accumulation of such biological liquids could lead to intraabdominal infection [1]. Bile and pancreatic secretions could also be toxic for surrounding tissue. In addition to eliminating dead space, drains allow early detection and quantification of intraabdominal hemorrhage and leakage of enteric suture lines [1]. Therefore, prophylactic drainage has gained popularity and acceptance throughout time as a simple, yet useful method to prevent complications after abdominal procedures. 

However, despite its advantages, placement of a prophylactic drain remains debated in many instances. While it does not offer clinically significant benefits for most abdominal surgical procedures (especially colorectal anastomoses) [1, 3-4, 6], its use remains controversial and individualized in other interventions, especially in instances involving the upper abdomen (pancreatic, biliary, and gastric procedures) where there remains a further demand for well-designed studies to clarify the value of prophylactic drainage [1]. 

Additionally, abdominal drains could be associated with complications, including hemorrhage, bacterial infection, hernia formation, foreign body reactions, and postoperative pain [1, 7-8]. Other less common, yet important, complications include the development of bronchoperitoneal fistula [9], bowel perforation from pressure necrosis [10], and small bowel evisceration [8]. 

Bowel obstruction is another very rare complication from intraabdominal drains. Shah et al. reported a case of a 78-year old male whose small bowel was twisted around a JP drain after undergoing low anterior resection with loop ileostomy for rectal cancer [11]. The obstructive phenomenon was described as a “maypole” effect. The patient underwent small bowel resection with primary anastomosis due to secondary intestinal necrosis. Additionally, Poon and colleagues [12] described a case where an abdominal drain led to small bowel obstruction after laparoscopic colectomy in an 82-year-old patient requiring reintervention. Another case of drain-associated intestinal obstruction after laparoscopic gastric bypass was reported by Rogers et al. [13]. 

To the best of our knowledge, this is the fourth case report ever published in the literature on intestinal obstruction caused by a surgical drain and the first such case following a hepato-pancreato-biliary procedure (Table 1).

**Table 1:** Reported cases of bowel obstruction from abdominal drains. JP: Jackson-Pratt

Author	Year	Initial Surgery	Findings	Treatment
Rogers [13]	2007	Laparoscopic Roux-en-Y gastric bypass for morbid obesity	Loop of bowel twisted around the abdominal drainage catheter	Nonsurgical removal of drain
Poon [12]	2009	Laparoscopic anterior resection	Herniation of the small bowel mesentery into the side holes of the silicon intra-abdominal drain leading to mechanical obstruction	Emergency laparoscopy with hernia reduction and drain removal
Shah [11]	2014	Low anterior resection with loop ileostomy for rectal cancer	Small bowel twisted around the drain	Small bowel resection with primary anastomosis due to intestinal necrosis
Present case	2019	Extended right hepatectomy, cholecystectomy, extrahepatic duct resection and lymphadenectomy, followed by a Roux-en-Y hepaticojejunostomy for Klatskin tumour	Proximal jejunum looped around the JP drain, close to the hepaticojejunostomy	Explorative laparotomy with removal of JP drain

On preoperative imaging, a close trajectory of the JP alongside the afferent loop was noted without clear evidence pointing out the drain as the cause of the intestinal obstruction. Indeed, the etiology of the obstruction on imaging was only noted retrospectively. This case serves as an example of how unlikely it is to suspect a drain as a cause of intestinal obstructions. 

All initial interventions of the four cases (including the following report) on intestinal obstruction from an abdominal drain were elective procedures. Of the four published cases on intestinal obstruction from an abdominal drain, only one case resolved nonoperatively [13]. Indeed, most cases required reintervention due to nonspecific findings on CT scan and/or worrisome postoperative presentations. This further emphasizes the importance of placing drains only when indicated, as undergoing a reoperation from iatrogenic complications increases the risk of patient morbidities. 

## Conclusions

Although therapeutic drainage of intraabdominal collections is beneficial, the role of prophylactic drainage following abdominal interventions remains controversial. There is currently growing evidence that prophylactic drain placement is not beneficial after different surgical interventions. Placement of prophylactic drains must be individualized. When placing them, the surgeon must take into consideration the possible related complications, including hemorrhage, infection, and rarely intestinal obstruction. Finally, with advances in surgical technique, easy access to antibiotics, radiological imaging and drainage, indications for routine prophylactic drains should be re-evaluated. 
